# Target Trial Emulation and Bias Through Missing Eligibility Data: An Application to a Study of Palivizumab for the Prevention of Hospitalization Due to Infant Respiratory Illness

**DOI:** 10.1093/aje/kwac202

**Published:** 2022-12-12

**Authors:** Daniel Tompsett, Ania Zylbersztejn, Pia Hardelid, Bianca De Stavola

**Keywords:** average causal effect, eligibility, missing data, multiple imputation, target trial emulation

## Abstract

Target trial emulation (TTE) applies the principles of randomized controlled trials to the causal analysis of observational data sets. One challenge that is rarely considered in TTE is the sources of bias that may arise if the variables involved in the definition of eligibility for the trial are missing. We highlight patterns of bias that might arise when estimating the causal effect of a point exposure when restricting the target trial to individuals with complete eligibility data. Simulations consider realistic scenarios where the variables affecting eligibility modify the causal effect of the exposure and are missing at random or missing not at random. We discuss means to address these patterns of bias, namely: 1) controlling for the collider bias induced by the missing data on eligibility, and 2) imputing the missing values of the eligibility variables prior to selection into the target trial. Results are compared with the results when TTE is performed ignoring the impact of missing eligibility. A study of palivizumab, a monoclonal antibody recommended for the prevention of respiratory hospital admissions due to respiratory syncytial virus in high-risk infants, is used for illustration.

## Abbreviations

ACE: average causal effect
CI: confidence interval
HTI: Hospital Treatment Insights database
MAR: missing at random
MCAR: missing completely at random
MI: multiple imputation
MNAR: missing not at random
RCT: randomized controlled trial
RSV: respiratory syncytial virus
TT: target trial

Randomized controlled trials (RCTs) are commonly used for estimating causal effects of point interventions. However, in many epidemiologic settings, an RCT may be infeasible or ethically nonviable. Hence, observational data are also used to compare effectiveness, with various strategies adopted to address the lack of randomization and indication bias, for example, by controlling for measured confounders. Analysis of observational data suffers from various additional sources of bias, such as selection bias, indication bias, and immortal time bias (1).

Target trial emulation aims to avoid some of these biases by adopting the design principles of RCTs. Individuals in an observational database, such as administrative health records, are selected according to a set of eligibility criteria that mirror those that would be used in an RCT (2). However, data on variables that determine eligibility are often incomplete, and as such not all participants of the target trial (TT) are identifiable from the observational database. It is typically advised to consider a different target trial with more complete eligibility criteria (1) or to exclude or censor individuals with missing data (3, 4). Missing data is often a source of bias when those excluded are systematically different from the observed (i.e.*,* if data are missing at random (MAR) or missing not at random (MNAR)) (5, 6). Although identified as a potential limitation, there is little work investigating the extent to which missing eligible data can impact the analysis of a target trial.

One solution is to impute missing eligibility prior to selection into a target trial. However, we could find only one precedent of imputation of eligibility criteria prior to the creation of a target trial in (7). More generally, multiple imputation (MI) of exclusion criteria in observational studies has been considered in a recent work (8) for validating error-prone confounders, but it remains an infrequently studied topic. We intend to bring attention to work of this kind to the context to target trial emulation.

In this paper we investigate biases in the average causal effect (ACE) of a point exposure, in a target trial with missing eligibility data. Our simulations consider realistic scenarios where the eligibility variables modify the true causal effect. We consider two strategies of analysis: 1) conditioning on variables that drive missingness eligibility, and 2) recovering the missing eligibility data via MI. A study of palivizumab, a monoclonal antibody for prevention of symptoms of severe respiratory syncytial virus (RSV) infection in high-risk infants, based on administrative hospital and pharmacy dispensing data is used to illustrate these alternative approaches.

## METHODS

### Setup

**Figure 1:**
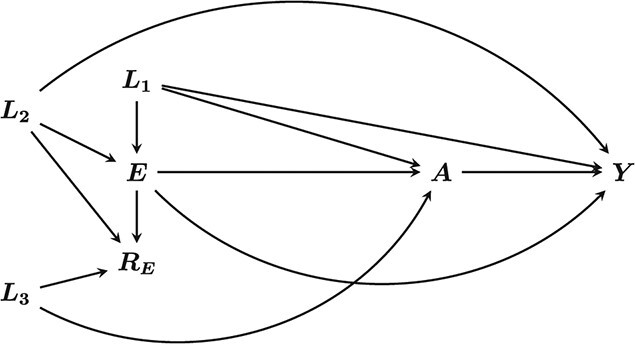
Directed acyclic graph of the assumed relationships between exposure, outcome, confounders, and data missingness indicator. *A* and *Y* are the exposure and outcome respectively. ${L}_1$ are confounders of the association between *A* and *Y*, with *E* the variable that determined eligibility for the target trial. ${L}_2$ and ${L}_3$ are drivers of missing data in $E$.

Consider the setting with a binary treatment $A$, end of study outcome $Y$, and confounding variables ${L}_1$ and $E$, where the latter determines eligibility. Suppose $E$ has informative missingness, with ${R}_E$ being an indicator of completeness (1 = complete, 0 = missing). Missingness in $E$ may be MAR, driven by variables that are not necessarily confounders, which we denote ${L}_2$ and ${L}_3$, or MNAR, if also driven by $E$ itself (9) (Figure 1). This is a typical setting, whereby ${L}_2$ and ${L}_3$ are separate causes of respectively $A$ and $Y$.

**Figure 2:**
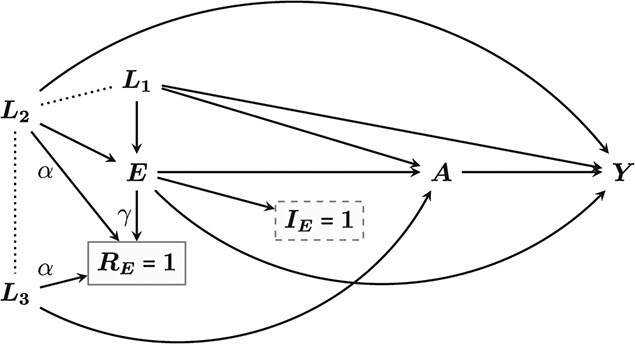
Directed acyclic graph of the assumed relationships between exposure (*A*), outcome (*Y*), and confounders (*L*), and the eligibility processes represented by the indicator ${I}_E$ plus the missing mechanism in $E$ represented by ${R}_E$. The solid and dashed boxes around these main indicators represent conditioning and the dotted lines represent spurious associations caused by this conditioning.

We emulate a TT where eligibility is defined by $E$ being greater than or equal to some value $e$. In practice $E$ may represent a set of variables, which determine an eligibility indicator variable ${I}_E$. The mechanism for inclusion is shown in Figure 2, represented by the box (indicating conditioning) surrounding ${I}_E=1$. It also shows the selection mechanism induced by restricting the TT to individuals with complete $E$, indicated by the box around ${R}_E=1$. We distinguish between:

- The source population, from which the TTs are derived
- The full eligibility TT ($ T{T}_{\mathrm{true}} $ ), containing all those who are eligible $ \left({I}_E=1\right) $
- The complete eligibility TT ($T{T}_{\mathrm{obs}}$), containing those who are complete and eligible, (${I}_E=1$ and ${R}_E=1$)

Our target estimand is the ACE of $A$ on $Y$ in $T{T}_{\mathrm{true}}$, defined as,

(1) \begin{equation*} \mathrm{AC}{\mathrm{E}}^{I_E=1}={\textrm{I}\!\textrm{E}}\left(Y(1)-Y(0)|{I}_E=1\right), \end{equation*}

where $E(Y(a))$ is the average value of $Y$, if the exposure $A$ were set to take the value of *a*, for *a* = 0,1 in the whole population. In reality, $T{T}_{\mathrm{true}}$ is not known, and thus ${\mathrm{ACE}}^{I_E=1}$ is approximated by the equivalent estimand from $T{T}_{\mathrm{obs}}$,

(2) \begin{equation*} {\mathrm{ACE}}^{I_E=1,{R}_E=1}={\textrm{I}\!\textrm{E}}\left(Y(1)-Y(0)|{I}_E=1,{R}_E=1\right). \end{equation*}

The ACE of a point exposure can be identified by invoking assumptions of no interference, counterfactual consistency, and conditional exchangeability (i.e.*,* no unmeasured confounding) (2).

### Sources of bias

If we attempt to estimate $AC{E}^{I_E=1}$ from an estimate of $AC{E}^{I_E=1,{R}_E=1}$ we would be prone to 2 sources of bias: collider bias and selection bias.

#### Collider bias.

The confounders ${L}_1$ and $E$ are common causes of exposure and outcome which need to be controlled for, while ${L}_2$ and ${L}_3$, the drivers of missingness, are not. However, when we condition on ${R}_E=1$, we create a spurious association between ${L}_2$ and ${L}_3$, which confounds the causal effect of $A$ on $Y$ via $A\to {L}_3\to {L}_2\to Y$ (Figure 2). This is a type of collider bias known as Berkson’s bias (10, 11), which must be removed by conditioning on either ${L}_2$ or ${L}_3$.

#### Selection bias.

When $E$ has informative missing data, the missing eligible (${I}_E=1,{R}_E=0$) contain information about $T{T}_{\mathrm{true}}$ that cannot be recovered by $T{T}_{\mathrm{obs}}$. This can result in selection bias when conducting an analysis on $T{T}_{\mathrm{obs}}$ if, for any reason, the causal effect of $A$ on $Y$ is different in the missing eligible, compared with the complete eligible.

By controlling for ${L}_1$, ${L}_2$, and $E$ we identify the causal effect,

(3) \begin{align*} \mathrm{AC}{\mathrm{E}}_{\left({L}_1,{L}_2,E\right)}^{I_E=1,{R}_E=1}={\textrm{I}\!\textrm{E}}(Y(1)-Y(0)|{L}_1={l}_1,{L}_2={l}_2,\notag\\E=e,{I}_E=1,{R}_E=1)\kern0.33em \forall \kern0.33em ({l}_1,{l}_2,e). \end{align*}

To find ${\mathrm{ACE}}^{I_E=1,{R}_E=1}$ we marginalize (average) ${\mathrm{ACE}}_{({L}_1,{L}_2,E)}^{I_E=1,{R}_E=1}$ over the distribution of ${L}_1$, ${L}_2$ and $E$ in $T{T}_{\mathrm{obs}}$.

If the effect of $A$ on $Y$ is modified by these confounders, then the value of ${\mathrm{ACE}}^{I_E=1,{R}_E=1}$ depends on the distribution of that confounder in $T{T}_{\mathrm{obs}}$.

Hence, since we cannot recover the distribution of the confounders in $T{T}_{\mathrm{true}}$, ${\mathrm{ACE}}^{I_E=1,{R}_E=1}$, obtained from $T{T}_{\mathrm{obs}}$, is a biased approximation of ${\mathrm{ACE}}^{I_E=1}$. In other words, the distribution of the confounders in $T{T}_{\mathrm{obs}}$, does not match that in $T{T}_{\mathrm{true}}$.

Suppose $E$ was a score capturing standards of hospital care. We might expect a treatment $A$ to be more effective on the outcome at higher standards of care. Now if hospitals of a low standard are more likely to have a missing score, then we would overrepresent eligible hospitals of higher standards in $T{T}_{\mathrm{obs}}$, and lead to a biased ACE.

Dealing with this bias requires recreating the joint distribution of exposure, outcome, and confounders of $T{T}_{\mathrm{true}}$, for example, using multiple imputation (12–14).

This bias has been discussed in the wider setting of “data-fusion” of multiple data sources (15), with identification of targeted causal effects involving knowledge of the distribution of the confounders in the “fused” population, as we discuss above. This type of bias has been referred to as an issue of “transportability” (15) or external validity to a different population (16). Our setting is created by missing information that precludes the identification of the target population. This could be viewed as an issue of internal validity of $T{T}_{\mathrm{obs}}$ itself, or of its external validity to $T{T}_{\mathrm{true}}$. The issue also has an impact on the generalizability of results to other populations.

### Strategies

We indicate possible strategies to address the above biases in the estimation of ${\mathrm{ACE}}^{I_E=1}$.

#### Strategy 1: ignoring missing eligibility.

In the setting of Figure 2, we fit an outcome regression model for $Y$ on $A$, controlling for ${L}_1$ and $E$ in the model, and then estimate ${\mathrm{ACE}}^{I_E=1,{R}_E=1}$ by marginalizing over their distribution in $T{T}_{\mathrm{obs}}$, as described in Aolin et al. (17).

#### Strategy 2: dealing with collider bias.

With this approach we fit an outcome regression model for $Y$ on $A$, controlling for ${L}_1$, $E$, and either ${L}_2$ or ${L}_3$ in order to block the path opened by conditioning on ${R}_E$, and then estimate ${\mathrm{ACE}}^{I_E=1,{R}_E=1}$ as in strategy 1.

If the estimand of interest is ${\mathrm{ACE}}^{I_E=1,{R}_E=1}$, then this strategy is sufficient to remove bias induced by missing eligibility data.

#### Strategy 3: dealing with collider and selection bias.

We specify an imputation model to predict the missing eligibility data in the source population. We impute $E$ in multiple copies of the source population and, from each, construct an imputed copy of $T{T}_{\mathrm{true}}$ using imputed eligibility criteria. We then control for ${L}_1$ and $E$ as in strategy 1 to estimate ${\mathrm{ACE}}^{I_E=1}$ in each of the copies, which are pooled using Rubin’s Rules (9).

##### Implementation.

The imputation step is as follows:

1. Specify an imputation model for the missing mechanism of $E$.
2. Generate $m$ copies of the source population and impute $E$ in each copy based on the imputation model.
3. Apply the eligibility criteria to each imputed data set to obtain $m$ emulated versions of $T{T}_{\mathrm{true}}$.
4. Estimate ${\mathrm{ace}}^{I_E=1}$ in each imputed $T{T}_{\mathrm{true}}$, controlling for ${L}_1$ and $E$ to obtain $m$ estimates of the causal effect of $A$ on $Y$, ${\widehat{ACE}}_m^{I_E=1}$.
5. Obtain Rubin’s pooled estimate of the target causal effect by taking the average over the $m$ imputed sets:
  $$ \begin{align*} {\widehat{ACE}}^{I_E=1}=\frac{1}{m}{\sum_{i=1}^m}{\widehat{ACE}}_m^{I_E=1} \end{align*}$$

To capture any suspected treatment effect heterogeneity, imputations are carried out separately for each value of $A$. Note that this technique requires $A$ be fully observed (18).

Giganti and Shepherd (8) highlight that excluding data relevant to inclusion in a study after MI leads to biased estimates of Rubin’s pooled estimate of the variance because of incongeniality between the imputation and outcome model. We hence consider confidence intervals using a percentile-based bootstrap.

##### Combining bootstrap and imputations.

We combine bootstrapping with MI using the “Boot-MI” methodology (19). This consists of the following steps:

1. Obtain $b$ bootstrap samples of the source population.
2. Apply steps 1–5 of the MI procedure above for each of the $b$ data sets, and obtain $b$ estimates of ${\widehat{ACE}}_b^{I_E=1}$.
3. A percentile-based bootstrapped confidence interval (CI) is then derived as the $\alpha \times {100}^{\mathrm{th}}$ and $(1-\alpha)\times {100}^{\mathrm{th}}$ percentiles of the ordered bootstrapped estimates.

We used single imputation $(m\!=\!1),$ which has been shown to have good statistical properties (20), and reduce computational burden (19, 20), nested within *b* = 1,000 bootstraps, which is at or above the typically recommended number (21).

##### Sensitivity analyses.

Imputation models for $E$ that allow for different mean values depending on $A$ could be used,

$$\begin{align*} {\textrm{I}\!\textrm{E}}\!\left(E|Y,A=a,{L}_1,{L}_2,{L}_3,{R}_E\right)={\beta}_0+{ \sum_{i=1}^3}{\beta}_i{L}_i+{\beta}_5Y+{\delta}_a{R}_E, \end{align*}$$

for *a* = 0,1.We use fully conditional specification (or multiple imputation by chained equations) using the “mice” package in R (R Foundation for Statistical Computing, Vienna, Austria) (13, 22) to impute the data. The parameters ${\delta}_a$ are MNAR sensitivity parameters. If MNAR is suspected, setting ${\delta}_a\ne 0$ shifts the imputed values of $E$ (separately for each $a$) by an amount that accounts for the effect of $E$ on its own missingness (23, 24). In practice, sensible ranges for ${\delta}_a$ are chosen, with the data imputed over these ranges.

## SIMULATIONS

We investigate strategies 1–3 by simulating data according to the structure of Figure 2. Specifically:

-
${L}_1$ , ${L}_2$, and ${L}_3$ are independent $N(0,1)$.
-
$ E $ is a normal variable dependent on $ {L}_1 $ and $ {L}_2 $:
  $$ E\sim N\!\left({L}_1+{L}_2,1\right). $$
- Eligibility is defined as ${I}_E=1$ if $E\ge 0$; ${I}_E=0$ otherwise. Hence, around 50% of the population is eligible.
- The missing mechanism of $E$ is expressed as a linear function of ${L}_2,$$ {L}_3 $, and $ E $:
  $$ \log\! \left(\mathrm{odd}{\mathrm{s}}_{R_E}\right)=\mu +\alpha {L}_2+\alpha {L}_3+\gamma E. $$
- The exposure $A$ is a binary variable, and generated in terms of the log-odds of exposure, expressed as a linear function of $ {L}_1 $$ {L}_3 $, and $ E $:
  $$ \log\! \left(\mathrm{odd}{\mathrm{s}}_A\right)=0.1{L}_1+0.5{L}_3+0.1E. $$
- Around 54% of individuals in the source population are exposed.
- The outcome $ Y $ is a normal variable that depends on exposure $ A $, eligibility $ E $, their interaction, and also on $ {L}_1 $ and $ {L}_2 $, with $ {L}_2 $ exercising a stronger impact than $ {L}_1 $:
  $$ Y\sim N\left(A+E+ AE+{L}_1+2{L}_2,1\right)\!. $$

The source population is of size $n=1,000$. We investigated strategies 1–3 at different values of $\mu$, $\alpha$, and $\gamma$, the parameters affecting ${R}_E$. Specifically, $\mu$ drives the percentage of missing completely at random (MCAR) missingness. $\alpha$ drives the strength of the MAR assumption, and the spurious association between ${L}_2$ and ${L}_3$, and $\gamma$ drives the strength of the MNAR mechanism, with positive values leading to a higher probability of larger values of $E$ being observed.

The parameter $\mu$ was set at 0 and 1.5, leading to severe (50%) and moderate (18%) MCAR missingness. $\alpha$ and $\gamma$ were set to range from 0 (no association) up to $\pm 0.4$. For each combination we carried out $l=1,000$ simulations for each of these scenarios using $b=\mathrm{1,000}$ bootstraps, reporting for each the average bias in the estimation $\big( AC{E}^{I_E=1,{R}_E=1}- AC{E}^{I_E=1}\big)$, its Monte Carlo error (MCE), root mean squared error (RMSE), and 95% coverage (25).

## RESULTS

### Observed and true target trial comparisons

Table 1 describes the characteristics of a set of single large simulations of $T{T}_{\mathrm{obs}}$ for different values of $\alpha$, $\gamma$, and $\mu$. We set $n=1,000,000$ to minimize random variation. The 3 missingness scenarios are MCAR ($\alpha =\gamma =0$), MAR ($\alpha \ne 0$ and $\gamma =0$), and MNAR ($\alpha \ne 0$ and $\gamma \ne 0$). The scenario when $E$ is not missing ($T{T}_{\mathrm{true}}$) is included for comparison.

**Table 1:** Summary Statistics of Simulated Variables in the Observed Target Trial for *n* = 1,000,000 for Selected Values of $\mu$, $\alpha$, and $\gamma$

$\boldsymbol{\mu}$	$\boldsymbol{\alpha}$	$\boldsymbol{\gamma}$	${P}_{R_E\mid {I}_E=1}$ ^**a**^	$\overline{E}$	${\overline{L}}_1$	${\overline{L}}_2$	${\overline{L}}_3$	${\boldsymbol{\rho}}_{(\mathbf{1},\mathbf{2})}$ ^**b**^	${\boldsymbol{\rho}}_{(\mathbf{2},\mathbf{3})}$
*No Missingness*									
0	0	0	1.00	1.38	0.46	0.46	0.0	−0.27	0.0
*MCAR*									
1.5	0	0	0.82	1.38	0.46	0.46	0.0	−0.27	0.0
0	0	0	0.50	1.38	0.46	0.46	0.0	−0.27	0.0
*MAR*									
1.5	0.4	0	0.83	1.40	0.45	0.51	0.07	−0.27	−0.02
1.5	0.2	0	0.83	1.39	0.45	0.49	0.04	−0.27	−0.01
1.5	−0.2	0	0.80	1.37	0.47	0.43	−0.04	−0.27	−0.01
1.5	−0.4	0	0.78	1.35	0.48	0.39	−0.08	−0.27	−0.02
0	0.4	0	0.54	1.44	0.42	0.60	0.17	−0.25	−0.03
0	0.2	0	0.52	1.41	0.44	0.53	0.09	−0.26	−0.01
0	−0.2	0	0.48	1.34	0.48	0.38	−0.10	−0.28	−0.01
0	−0.4	0	0.46	1.31	0.50	0.30	−0.20	−0.28	−0.03
*MNAR*									
1.5	0.4	0.4	0.88	1.43	0.46	0.51	0.05	−0.26	−0.02
1.5	0.2	0.2	0.86	1.42	0.46	0.49	0.03	−0.26	−0.01
1.5	−0.2	−0.2	0.75	1.31	0.45	0.40	−0.05	−0.29	−0.01
1.5	−0.4	−0.4	0.67	1.20	0.44	0.32	−0.12	−0.31	−0.04
1.5	0	−0.4	0.71	1.25	0.42	0.42	0.00	−0.30	0.00
1.5	0.4	−0.4	0.42	1.23	0.34	0.55	0.22	−0.29	−0.02
1.5	−0.4	0.4	0.58	1.49	0.55	0.39	−0.16	−0.24	−0.02
1.5	0.2	−0.2	0.46	1.31	0.40	0.50	0.11	−0.28	−0.01
1.5	−0.2	0.2	0.55	1.45	0.51	0.42	−0.09	−0.26	−0.00
0	0.4	−0.4	0.74	1.31	0.40	0.50	0.10	−0.28	−0.01
0	−0.4	0.4	0.85	1.42	0.49	0.43	−0.06	−0.26	0.00
0	0.2	−0.2	0.78	1.35	0.44	0.48	0.04	−0.27	0.00
0	−0.2	0.2	0.84	1.40	0.48	0.44	−0.03	−0.26	0.00
0	0.4	0.4	0.66	1.54	0.48	0.60	0.13	−0.25	−0.04
0	0.2	0.2	0.59	1.49	0.47	0.55	0.08	−0.25	−0.01
0	−0.2	−0.2	0.41	1.22	0.45	0.33	−0.11	−0.31	−0.01
0	−0.4	−0.4	0.34	1.07	0.44	0.19	−0.24	−0.34	−0.04
0	0	−0.4	0.37	1.13	0.38	0.38	0.00	−0.32	0.00

Abbreviations: MAR, missing at random; MCAR, missing completely at random; MNAR, missing not at random.

^a^ Note that ${P}_{R_E\mid {I}_E=1}=\mathit{\Pr}({R}_E=1|{I}_E=1)$ is a measure of the number of missing eligible participants.

^b^ ${\rho}_{1,2}=\mathrm{Corr}({L}_1,{L}_2)$ ; ${\rho}_{2,3}=\mathrm{Corr}({L}_2,{L}_3)$.

When the mechanism is MCAR, the means and correlations of relevant variables are not affected. When the mechanism is MAR, they depart from those found in $T{T}_{\mathrm{true}}$: When $\alpha >0$, individuals in $T{T}_{\mathrm{obs}}$ have larger mean values for $E$, ${L}_2$, and ${L}_3$ than in $T{T}_{\mathrm{true}}$. This is because $\alpha$ leads to individuals with larger values for ${L}_2$ and ${L}_3$ being more likely to be observed, shifting upward their distributions, and by extension, the distribution of $E$. When $\alpha$ is negative, the opposite is true. These biases are more noticeable at $\mu =0$ due to the greater proportion of missing individuals.

Under MNAR, setting $\gamma >0$ makes higher values of $E$ more likely to be observed in $T{T}_{\mathrm{obs}}$, with the opposite occurring when $\gamma <0$, leading to shifts in the distributions for $E$, ${L}_2$, and ${L}_3$ similar to what occurs with $\alpha$.

The combined impact of $\alpha$ and $\gamma$ varies. When both are of the same sign, their impacts compound and strengthen the corresponding shifts in distribution. When they are of opposite sign, their impacts partially offset one another.

The shifts in distribution for ${L}_1$ are complicated, shifted downward when $\alpha >0$ but shifted upward when $\gamma >0$. This is due to a complicated relationship between the spurious negative ${L}_1-{L}_2$ association (caused by conditioning on ${I}_E$), driving a downward shift in ${L}_1$ with higher values of ${L}_2$, and the positive ${L}_1-E$ association, driving an upward shift with higher values of $E$.

### Strategies

For strategies 1 and 2, bias in estimation of ACE increased with higher values of α and γ, and was worse when μ = 0 (Tables 2 and 3). This is due to having to average over the distribution of the confounders to estimate $AC{E}^{I_E=1}$. The size and direction of this bias is nearly identical to the shift in the distribution of $E$ observed in Table 1. This is because effect modification by $E$ has effect size equal to 1.

**Table 2:** Results of Applying the 3 Strategies to Data Generated Under Different Scenarios, With $\mu =1.5$ and $n=1,000$

**Strategy**	$\boldsymbol{\alpha}$ ^**a**^	$\boldsymbol{\gamma}$	**Bias**	**Coverage**	**RMSE**	**MCE**
*No Missingness*						
1	0	0	0.00	95.0	0.00	0.01
*MCAR*						
1	0	0	0.01	94.4	0.17	0.01
2			0.01	93.9	0.1	0.00
3			−0.01	95.4	0.17	0.01
*MAR*						
1	−0.4	0	−0.04	94.8	0.20	0.01
2			−0.03	93.4	0.14	0.00
3			0.00	95.9	0.17	0.01
1	0.4	0	0.02	94.2	0.17	0.01
2			0.03	93.1	0.10	0.00
3			−0.01	95.3	0.17	0.01
*MNAR*						
1	0.4	0.4	0.05	93.9	0.17	0.01
2			0.06	91.6	0.14	0.00
3			−0.01	95.2	0.05	0.01
1	0.2	0.2	0.04	94.6	0.17	0.01
2			0.04	92.2	0.10	0.00
3			−0.01	95.4	0.17	0.01
1	−0.2	−0.2	−0.07	93.1	0.20	0.01
2			−0.07	89.9	0.14	0.00
3			0.01	95.7	0.05	0.01
1	−0.4	−0.4	−0.19	82.4	0.26	0.01
2			−0.17	70.4	0.22	0.00
3			0.00	96.9	0.20	0.01

Abbreviations: MAR, missing at random; MCAR, missing completely at random; MCE, Monte Carlo error; MNAR, missing not at random; RMSE, root mean square error; $T{T}_{\mathrm{obs}}$, target trial emulated from observed data; *TT*_true_, the full eligibility target trial, containing all those who are eligible.

^a^ Average size of $T{T}_{\mathrm{obs}}$ for the 7 settings of $\alpha$ and $\gamma$ are *n* = 410, 387, 415, 442, 430, 374, and 332 respectively. Average size of $T{T}_{\mathrm{true}}$ is 500. Note that the average causal effect $AC{E}^{I_E=1}$ was calculated from a single simulation with $n=1,000,000$ and was estimated at 2.386.

**Table 3:** Results of Applying the 3 Strategies to Data Generated Under Different Scenarios, With $\mu =0$ and $n=1,000$

**Strategy**	$\boldsymbol{\alpha}$ ^**a**^	$\boldsymbol{\gamma}$	**Bias**	**Coverage**	**RMSE**	**MCE**
*No Missingness*						
1	0	0	0.00	95.0	0.00	0.01
*MCAR*						
1	0	0	0.01	95.0	0.24	0.01
2			0.01	94.6	0.14	0.00
3			0.00	97.9	0.22	0.01
*MAR*						
1	−0.4	0	−0.09	93.2	0.26	0.01
2			−0.07	91.7	0.17	0.00
3			−0.01	97.9	0.24	0.01
1	0.4	0	0.05	94.3	0.22	0.01
2			0.07	92.8	0.14	0.00
3			0.00	97.6	0.22	0.01
*MNAR*						
1	0.4	0.4	0.16	86.3	0.26	0.01
2			0.17	73.0	0.22	0.00
3			−0.00	97.3	0.20	0.01
1	0.2	0.2	0.12	90.4	0.24	0.01
2			0.12	85.3	0.17	0.00
3			0.00	97.6	0.22	0.01
1	−0.2	−0.2	−0.17	90.0	0.30	0.01
2			−0.15	83.3	0.22	0.00
3			0.00	98.2	0.07	0.01
1	−0.4	−0.4	−0.33	75.3	0.42	0.01
2			−0.31	53.4	0.34	0.01
3			0.01	98.7	0.17	0.01

Abbreviations: MAR, missing at random; MCAR, missing completely at random; MCE, Monte Carlo error; MNAR, missing not at random; RMSE, root mean square error; $T{T}_{\mathrm{obs}}$, target trial emulated from observed data; *TT*_true_, the full eligibility target trial, containing all those who are eligible.

^a^ Average size of $T{T}_{\mathrm{obs}}$ for the 7 settings of $\alpha$ and $\gamma$ are *n* = 250, 228, 271, 328, 294, 206, and 172 respectively. Average size of $T{T}_{\mathrm{true}}$ is 500. Note that the average causal effect $AC{E}^{I_E=1}$ was calculated from a single simulation with $n=1,000,000$ and was estimated at 2.386.

The impact of collider bias induced by $\alpha$ is negligible, as shown by the small differences in bias for strategies 1 and 2. The root mean squared error is smaller for strategy 2 but has more undercoverage, possibly because it involves averaging ${L}_2$, which also has a shifted distribution.

Table 1 implies that had ${L}_2$ been the effect modifier rather than $E$, strategies 1 and 2 would have shown more bias under the MAR assumption. This is investigated in Web Appendix 1 and Web Table 1 (available at https://doi.org/10.1093/aje/kwac202; additional supporting information can be found in Web Tables 2–4).

Strategy 3 shows unbiased estimates (within Monte Carlo error) in all cases, indicating a successful recovery of the causal effect in $T{T}_{\mathrm{true}}$. The CIs, however, display overcoverage, particularly when a large fraction of the eligible are missing.

Selection bias appears to increase under the following conditions:

- Larger numbers of missing eligible individuals
- Larger values of $\alpha$ and $\gamma$, the drivers of missingness
- A stronger effect modification of the causal effect of $A$ on $Y$ by $E$ (or any variables related to $E$)

With fewer eligible participants lost to missingness, there is less missing data to drive a differentiation in the distributions of $E$ in $T{T}_{\mathrm{obs}}$ and $T{T}_{\mathrm{true}}$, which is why bias decreased when $\mu$ was larger, and the number of missing eligible participants decreased. None of these features are likely to be known in advance.

When $E$ was MNAR, imputation was carried out with the correct values of the sensitivity parameters ${\delta}_0,{\delta}_1$. This was to demonstrate that, all other biases (including a misspecified imputation model) accounted for, strategy 3 can eliminate the biases described above in Sources of Bias when $E$ is MNAR. This is unlikely to be possible in reality; hence, in Web Appendix 2 we repeat specific MNAR simulations of Table 3 assuming a MAR imputation model $({\delta}_0,{\delta}_1)\!=(0,0)$, which shows notable bias. This highlights that, in practice, MNAR imputation is an exploratory technique, and careful considerations must be made to choose informative values of ranges for ${\delta}_0$ and ${\delta}_1$ to investigate (23, 24). A realistic application of strategy 3 is shown in the case study.

In summary, strategy 3 is necessary in the case that missing data are noticeably MAR or MNAR. If that is not the case, a user may prefer the simpler strategies 1 and 2. Strategy 2 is the most precise, if this is preferred by the user, but one must account for the possibility of undercoverage if a CI is sought.

## CASE STUDY: EFFECT OF PALIVIZUMAB ON INFANT HOSPITAL ADMISSION

RSV is a major cause of acute lower respiratory tract infection in infants, with RSV bronchiolitis responsible for 40,000 hospital admissions annually in England (26). Palivizumab is licensed for passive immunization to prevent RSV in premature infants with congenital heart disease or chronic lung disease. Due to its high cost, palivizumab is typically recommended to more select groups of high-risk infants than those in clinical trials, with limited data on real-world effectiveness (27). Hence, analysis by a selective emulated trial is of interest.

An observational cohort of infants potentially eligible for palivizumab treatment in England has been developed (27), using the Hospital Treatment Insights database (HTI), which links pharmacy dispensing records from 43 acute hospitals in England, and hospital records from Hospital Episode Statistics (HES). This cohort details infants born between January 1, 2010, and December 31, 2016, with follow-up data on palivizumab prescriptions and hospital admission up to their first year of life. HTI is maintained by IQVIA (https://www.iqvia.com/).

**Figure 3:**
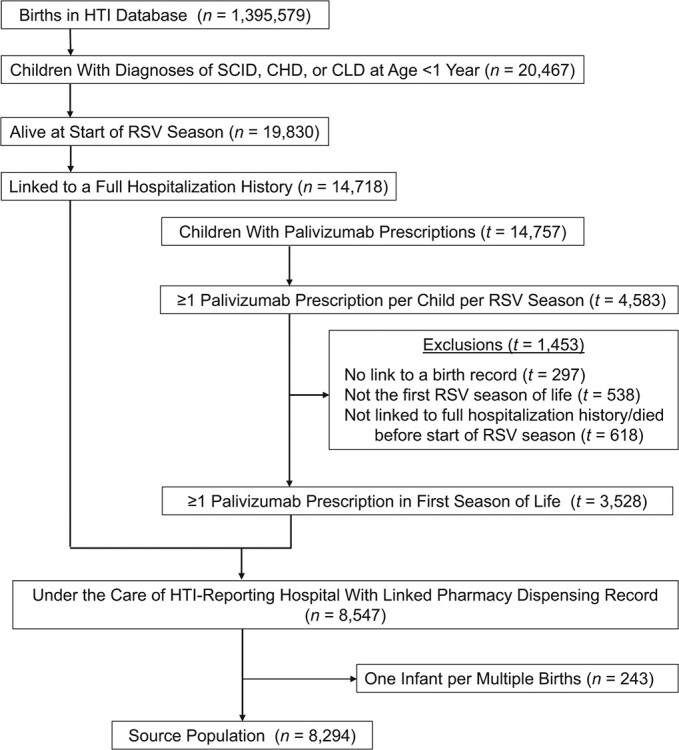
Derivation of the source population for the IQVIA (https://www.iqvia.com/) cohort; infants born in England between January 1, 2010, and December 31, 2016, with linked Hospital Episodes Statistics and prescription data. Note that the palivizumab prescriptions database contains a separate but overlapping population from those in the Hospital Treatment Insights database (HTI). Thus, this population is denoted by $t$ until linked to individuals in the HTI population ($n$). CHD, congenital heart disease; CLD, chronic lung disease; RSV, respiratory syncytial virus; SCID, severe combined immunodeficiency.

**Figure 4:**
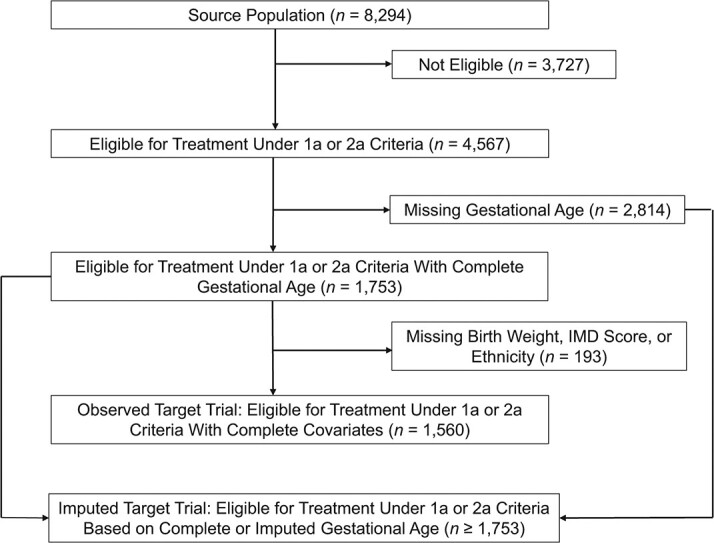
Derivation of the complete records target trial and of the imputed target trials of palivizumab treatment; infants born in England between January 1, 2010, and December 31, 2016, with linked Hospital Episodes Statistics and prescription data who were eligible to receive treatment under 1a and 2a criteria. Note that the exact size of the imputed target trial is unknown, and depends on the imputed data, but must be at least of size 1,753 (those with complete eligible data who qualify).

This cohort identifies a source population of 8,294 high-risk infants, defined as having congenital heart disease or chronic lung disease, under care of an HTI-reporting hospital, alive at the start of their first RSV season (October 1 to March 31), with a full linked hospital admission history. This is shown in the cohort flow charts of Figures 3 and 4.

Infants in the source population were considered eligible for the TT if they had a diagnosis of congenital heart disease or chronic lung disease and met additional eligibility criteria based on gestational age and chronological age at start of RSV season and, specifically, those who met 1a or 2a criteria for recommendation of treatment by palivizumab in Chapter 27a in the Green Book (28) (Web Table 3). Gestational age, however, is missing for 2,814 (34%) infants in the source population. As a result, the eligibility of many children cannot be identified.

### Target trial emulation

The emulated target trial protocol is detailed in Web Appendix 3 and Web Table 2. We define $T{T}_{\mathrm{obs}}$ to include all eligible individuals with complete eligibility data on gestational age, birth weight, Index of Multiple Deprivation score, and ethnicity. This led to a trial of 1,560 infants. We also aimed to recover $T{T}_{\mathrm{true}}$ by imputing missing gestational age in the high-risk cohort. This corresponds to using strategies 2 and 3, respectively.

We are interested in the effect of any palivizumab prescription on RSV-related hospital admission in infants during their first RSV season of life. A full course of treatment by palivizumab requires up to 5 monthly doses during RSV season. As we could not determine adherence to treatment from the HTI data, we define a simplified exposure as a binary indicator of having been prescribed at least 1 dose of palivizumab in their first RSV season of life. Infants are identified in the first month of life for treatment, and it is typically administered in outpatient clinics, not when hospitalized for RSV. Our outcome is a binary indicator of having been hospitalized for an RSV-related condition during their first RSV season of life.

Our target estimand is the ACE of palivizumab prescription on RSV-related hospital admission in $T{T}_{\mathrm{true}}$, expressed as the average difference in absolute risk of hospital admission (the intent to treat (ITT) effect).

To balance the confounders in the treated and untreated, we fitted a model for the propensity of receiving palivizumab, including gestational age, age at start of RSV season, Index of Multiple Deprivation quintiles, sex, ethnicity, year of birth, diagnosis of congenital heart disease or chronic lung disease (or both), and other comorbidities. The resultant propensity scores showed reasonable overlap in the treated and untreated (Web Figure 1). Mean differences between treated and untreated, adjusted for inverse probability of weighting by propensity score, were within 0.1, indicating good confounder balance.

We fitted 2 different outcome models, a logistic regression model of hospital admission against treatment with inverse probability weight of being treated (IPTW), corresponding to a marginal structural model (MSM) (29), and a second where we controlled for the propensity score and all confounders directly in the outcome model, similar to those in 2-stage g-estimation of structural nested mean models (SNMMs) (30). The ACE is calculated by estimating potential outcomes via the “data stacking” method of (17).

Continuous gestational age is imputed in the treated and untreated arms separately (to account for any interaction between gestational age and palivizumab) using a MNAR imputation model that includes all the variables of the propensity score model, plus the outcome and birth weight. Birth weight is not included in the outcome model due to collinearity with gestational age. There are thus 2 sensitivity parameters: ${\delta}_1$ for exposed and ${\delta}_0$ for unexposed. We assert that infants with missing gestational age may have higher mortality, implying a shorter gestation (31). Hence we performed the analysis setting ${\delta}_1$ and ${\delta}_0$ to either 0 (MAR), or −4 (MNAR).

Based on recommendations in Tompsett et al. (23), rather than compare the ACE directly with ${\delta}_0$ and ${\delta}_1$, which are difficult to interpret physically, we estimated from the imputed data the mean gestational age in treated and untreated infants to contrast against the results.

Missing birth weight, Index of Multiple Deprivation score, and ethnicity were imputed alongside gestational age using MICE. We report the results in Tables 4 and 5 below.

**Table 4:** Estimate of the Average Causal Effect for the Palivizumab Case Study Obtained Using Strategies 2 and 3, Using an Outcome Model Controlled for Confounders and Propensity Score

**Sensitivity Parameters**	**Trial Size**	$\widehat{ACE}$ ^**a**^	$\widehat{ACE}$ **, %**	**95% CI**	**Mean Gestational Age, Treated**	**Mean Gestational Age, Untreated**
$T{T}_{\mathrm{obs}}$						
N/A	1,560	−0.003	−0.3%	−0.05, 0.05	26.5	27.2
$T{T}_{\mathrm{imp}}$						
(0,0)	2,643	−0.002	−0.2%	−0.04, 0.04	26.9	27.7
(−4,0)	3,659	−0.010	−1.0%	−0.04, 0.03	26.9	25.5
(0,−4)	2,985	0.013	1.3%	−0.03, 0.05	24.2	27.7
(−4,-4)	3,964	0.006	0.6%	−0.02, 0.04	24.2	25.5

Abbreviations: ACE, average causal effect; CI, confidence interval; N/A, not applicable; $T{T}_{\mathrm{imp}}$, target trial emulated from observed and imputed data; $T{T}_{\mathrm{obs}}$, target trial emulated from observed data.

^a^ The ACE is expressed as a risk difference both in absolute value and in percentage risk difference The sensitivity parameters are listed in order $({\delta}_0,{\delta}_1)$. With sensitivity parameter $T{T}_{\mathrm{imp}}$(0,0), the data are assumed missing at random. In all other cases for $T{T}_{\mathrm{imp}}$it is assumed missing not at random. This is not applicable for $T{T}_{\mathrm{obs}}$, which has complete data.

**Table 5:** Estimated Average Causal Effect for the Palivizumab Case Study Obtained Using Strategies 2 and 3, Using an Inverse Probability Weighted Outcome Model

**Sensitivity Parameter**	**Trial Size**	$\widehat{ACE}$ ^**a**^	$\widehat{ACE}$ **, %**	**95% CI**	**Mean Gestational Age, Treated**	**Mean Gestational Age, Untreated**
$T{T}_{\mathrm{obs}}$						
N/A	1,560	−0.010	−1.0%	−0.06,0.04	26.5	27.2
$T{T}_{\mathrm{imp}}$						
(0,0)	2,643	−0.001	−0.1%	−0.04,0.04	26.9	27.7
(−4,0)	3,659	−0.031	−3.1%	−0.08,0.01	26.9	25.5
(0,−4)	2,985	0.023	2.3%	−0.03,0.07	24.2	27.7
(−4,-4)	3,964	0.011	(1.1%)	−0.03,0.05	24.2	25.5

Abbreviations: ACE, average causal effect of treatment; CI, confidence interval; N/A, not applicable; $T{T}_{\mathrm{imp}}$, target trial emulated from observed and imputed data; $T{T}_{\mathrm{obs}}$, target trial emulated from observed data.

^a^ The ACE is expressed as a risk difference both in absolute value and in percentage risk difference The sensitivity parameters are listed in order $({\delta}_0,{\delta}_1)$. With sensitivity parameter $T{T}_{\mathrm{imp}}$(0,0), the data are assumed missing at random. In all other cases for $T{T}_{\mathrm{imp}}$ it is assumed missing not at random. This is not applicable for $T{T}_{\mathrm{obs}}$, which has complete data.

### Results

Analysis of $T{T}_{\mathrm{obs}}$ suggests that treatment by at least 1 dose of palivizumab has little effect on the risk of being hospitalized, indicated by an ACE of $-0.003$ using a propensity score–conditioned outcome model, and $-0.01$ under inverse probability weighting (a 0.3% or 1.0% lower risk of hospital admission). When imputing the TT under MAR we observed a 0.1% and 0.2% lower risk of hospital admission, respectively. Under MNAR there is a more noted effect of palivizumab, ranging from −1.0% to 1.3% using an outcome model controlled for the propensity score, and −3.1% to 2.3% using inverse probability weighting.

The imputation model implies a high number of missing eligible participants, with over 1,000 more individuals under the MAR imputed trial, and up to nearly 2,500 more under MNAR.

When ${\delta}_0$ was set to $-4$, this led to a reduction in average gestational age in the untreated by 2.2 weeks. In this case there was stronger reduction in risk of hospital admission when treated. When ${\delta}_1$ was set to $-4$, the average gestational age in the treated was reduced by 2.7 weeks and there was an increasing risk of hospital admission under treatment.

No estimate was found to be significant based on a 95% CI. Despite there being a clear change in the distribution of gestational age under MNAR conditions, and a large number of missing eligible, there is only weak evidence of selection bias in this study. This implies that gestational age only weakly modifies the effect of palivizumab on hospital admission.

The implication is that receiving at least 1 dose of palivizumab appears to have little effect on hospital admission, and the results are robust to changes in the missing data assumption.

## DISCUSSION

In this paper we bring to light notable sources of bias in target trial emulation, emanating from ignoring missing eligible data. We explored one means to analyze a TT combined with multiple imputation of eligibility criteria prior to selection. We demonstrated via simulation that an imputed TT can eliminate sources of selection and collider bias, improve the sample size of a TT, and allow users to investigate sensitivity to changes in the assumptions of the missing eligible data on effect size.

An imputed TT of the effect of receiving at least 1 dose of palivizumab on RSV-related hospital admission indicated that a significant number of infants with missing gestational age were eligible, although any selection bias in this case was small.

We identified characteristics of the data that determine the size of selection bias, namely the strength of the MAR or MNAR mechanism, the number of missing eligible individuals and the size of the effect modification. None of these characteristics can be calculated from the source population but could be inferred using external linked data sets. This selection bias can occur if any variable related to eligibility is an effect modifier. We showed in Web Appendix 1 that when ${L}_2$ was the effect modifier, strong selection bias was identified when $E$ was MAR.

A limitation of this method is the tendency of CIs to overcover. The Boot-MI method (19) is computationally intensive, and thus one should expect an analysis to take several hours even with cluster computing methods. Hence we constructed CIs using a percentile bootstrap with just single imputation. However, single imputation lends itself to overcoverage (19). In Web Appendix 2, we applied strategy 3 using MI with $m=5$, which demonstrates improved coverage. One alternative would be to investigate the corrected Rubin’s pooled variance of $AC{E}^{I_E=1}$ suggested in Giganti and Shepherd (8). However, obtaining accurate confidence interval estimates in this way for the ACE using MI requires complex methods (32–34).

Instead of MI, we could consider using inverse probability weighting to address the bias caused by missingness in *E* (35). We investigated this method in Web Appendix 2 and found that it did not correct the bias. Another possible alternative is to utilize the work in Bareinboim and Pearl (15), by inferring or presuming the distribution of the confounders in $T{T}_{\mathrm{true}}$ and standardizing the conditional ACE estimated in $T{T}_{\mathrm{obs}}$, but it would be a considerable challenge.

It is also worth noting that using strategy 2, and targeting the causal effect in those with complete records, may be a pragmatic choice if the expected selection bias is limited and the source population is cumbersome.

Data on palivizumab prescriptions and adherence were limited, and this had an impact on the quality of conclusions that could be made. Clinical colleagues reassure us that children hospitalized with RSV would not be issued palivizumab, protecting from reverse causation. However, other issues, such as confounding by indication, cannot be discounted. Limitations of the diagnostic data also meant a slight inflation of our definition of the eligible population because some of the diagnoses may include less severe diseases than those listed in the Green Book (28).

## Supplementary Material

Web_Material_kwac202
