# Supplementary material for: Target Trial Emulation and Bias Through Missing Eligibility Data: An Application to a Study of Palivizumab for the Prevention of Hospitalization Due to Infant Respiratory Illness
Source: Am J Epidemiol. 2022 Dec 12;192(4):600–11. doi: 10.1093/aje/kwac202 (PMC10089079; doi:10.1093/aje/kwac202)
Supplement: Web_Material_kwac202 [file web_material_kwac202.zip › kwac202 Tompsett Web Material Final.pdf]

Web Appendix

Target Trial Emulation and Bias Through Missing Eligibility Data: An Application to a Study of Palivizumab for the Prevention of Hospitalization due to Infant Respiratory Illness

Daniel Tompsett[1], Ania Zylbersztejn[2], Pia Hardelid[3], and Bianca De Stavola[4]

[1]Correspondence to Dr Daniel Tompsett, Population Policy and Practice Department, UCL GOS Institute of Child Health, United Kingdom, 30 Guilford Street, London, WC1N 1EH, e-mail: d.tompsett@ucl.ac.uk

November 8, 2022

Contents

1 Web Appendices **3**

1.1 Web Appendix 1: Effect Modification By L_2 . . . 3

1.2 Web Appendix 2: Alternative Methods: Multiple Imputation, IPW, and MAR Imputation Model for MNAR . . . 3

1.3 Web Appendix 3: Trial Protocol and Eligibility Criteria . . . 5

1.4 Web Appendix 4: R Code Requests . . . 5

2 Web Tables **6**

2.1 Web Table 1 . . . 6

2.2 Web Table 2 . . . 7

2.3 Web Table 3 8

2.4 Web Table 4 9

3 Web Figures **10**

3.1 Web Figure 1 10

1 Web Appendices

1.1 Web Appendix 1: Effect Modification By L_2

In this appendix, we repeat a shorthand of the simulations of Section 4 under the following differences.

- The data is simulated as before, except that L_2 now modifies the effect of A on Y, simulated as

$$Y \sim N(A + E + AL_2 + L_1 + 2L_2, 1).$$

- As we focus on the bias. We perform the analysis on a single large dataset of size $n = 1000000$.

- We will compare strategies 1 to 3. The outcome model from which the ACE is calculated for strategy 1 controls for L_1 and E, and for strategies 2 and 3 also controls for L_2, and the $L_2 : A$ interaction. The imputation model for E is the same as Section 4.

- We focus on a specific selection for values of α, γ and μ.

The results in Web Table 1 indicate that strategies 1 and 2 continue to show bias when L_2 is set to the effect modifier. Notably, the bias under MAR conditions is larger than when E is the effect modifier. The bias in strategy 1 is slightly more skewed in the negative direction compared to strategy 2 due to collider bias. This bias is resolved in strategy 3 as in tables 2 and 3.

1.2 Web Appendix 2: Alternative Methods: Multiple Imputation, IPW, and MAR Imputation Model for MNAR

In this appendix we will briefly assess two modifications to our methods to potentially account for the selection bias observed in the paper. We will apply this to two simulation setups as given in section 4. One where $\mu = 0$, $\alpha = 0.4$ and $\gamma = 0$, and another where $\mu = 0$, $\alpha = 0.4$ and $\gamma = 0.4$.

1. 3 $(m = 5)$: Perform Strategy 3, but with multiple imputed datasets. Specifically, we will $set m = 5$.

2. 2 (IPW): Perform Strategy 2, but weight the outcome model using Inverse Probability Weights of being observed.

3. 3 (MAR): Perform Strategy 3 with E being MNAR ($\gamma \neq 0$), but impute using a MAR imputation model.

The second method is performed using the IPW weight as described in [1]. We first fit to the source population a logistic regression propensity score model for being observed ($R_E = 1$) as

$$logit(\mathbb{E}(R_E|L1, L2, L3)) = \zeta_0 + \zeta_1 L_1 + \zeta_2 L_2 + \zeta_3 L_3.$$

From which we predict a propensity score prs_{R_E} for the probability of being observed for each individual. We then derive TT_{obs} and perform strategy 2, but weight the outcome model used to estimate ACE$^{I_E=1,R_E=1}$ with weights $\frac{1}{prs_{R_E}}$.

As in section 4, we use $l = 1000$ simulated datasets of source population size $n = 1000$, with $b = 1000$ bootstraps. The results are shown in Web Table 4.

Web Table 4 suggests that IPW has not addressed the selection bias found in strategy 2, displaying only marginally improved bias and coverage. It is expected that IPW would not be effective when E is MNAR, as in this instance the propensity score model for R_E depends on E itself (which cannot be included in the model). However bias remains unaddressed when E is MAR. This may be due to the manner in which ACE$^{I_E=1,R_E=1}$ is calculated. Specifically, the weighted outcome model does not appear to address the issue of marginalising over the distribution of E which remains biased.

Using strategy 3 with $m = 5$ multiply imputed data sets fares better however. The ACE remains unbiased, and in particular, results shows slightly better precision (lower RMSE) than strategy 3 with $m = 1$. Whilst there is still an issue of over-coverage, it is notably closer to nominal than with strategy 3. The drawback is that these simulations were extremely computationally intensive, each taking over 100 hours to complete. Furthermore, different simulation setups to those used in this paper may not demonstrate the same improvement of coverage with increasing m, For example, target trials with more complicated eligibility criteria may have imputation and outcome models have more serious issues with incongeniality. However the re-

sult suggest that applying strategy 3 with MI may improve the coverage if it is computationally viable.

Finally applying strategy 3 when E is MNAR using a MAR imputation model shows noticeable bias and coverage issues. This is expected, as the imputation is mis-specified. Specifically the size of this bias will depend on the size of the mis- specification of the sensitivity parameters δ_a compared to their true values. When imputing under MAR we essentially set $\delta_a = 0$, whilst the true absolute values for δ_a ranged between $0.04 - 0.2$, and hence we note some small but noticeable bias. In this specific simulation setup, the bias caused by using a MAR imputation model (0.08) is smaller than when using strategy 2 (0.17). However, this may not hold true in general. In cases where the true values for δ_a deviate further from zero, one may expect strategy 3 (MAR) to begin to perform worse than 2. Hence we do not make any specific conclusions with regards to comparing these strategies. Instead we highlight the importance of recognizing when a missing variable may be MNAR, and choosing informative ranges for δ_a, that include their true unknown values, by using expert opinion or external datasets. What Tables 2 and 3 show however is that a sensitivity analysis can be conducted free of collider bias, which can also solve the issue of selection bias.

1.3 Web Appendix 3: Trial Protocol and Eligibility Criteria

The Target Trial protocol is detailed in Web Table 2 below.

1.4 Web Appendix 4: R Code Requests

The R code used to perform the simulations and case study are available on request. Please contact Dr Daniel Tompsett at `d.tompsett@ucl.ac.uk`

2 Web Tables

2.1 Web Table 1

Strategy	α	γ	μ	Bias
1	0	0	0	0.00
2	0	0	0	0.01
3	0	0	0	0.00
1	-0.4	0	0	-0.19
2	-0.4	0	0	-0.16
3	-0.4	0	0	0.00
1	0.4	0	0	0.13
2	0.4	0	0	0.14
3	0.4	0	0	0.00
1	-0.4	-0.4	0	-0.27
2	-0.4	-0.4	0	-0.26
3	-0.4	-0.4	0	0.00
1	0.4	0.4	0	0.12
2	0.4	0.4	0	0.13
3	0.4	0.4	0	0.00

Web Table 1 - Simulation results for the ACE with effect modification due to L_2 for a single large simulation. Note that the true value of the ACE is 1.46.

2.2 Web Table 2

Trial Component	Target Trial Protocol
Eligibility Criteria	Infants in source population (Figure 3):<br>• Meet criteria 1a or 2a as listed in Chapter 27a of the Green book<br>Based on complete, or possibly imputed gestational age.<br>• Born in NHS hospital between 1st Jan 2010 and 31st Dec 2016.<br>• Under care of HTI hospital trust.
Treatment Strategy	• 1: Recieved at least one dose of Palivizumab during RSV season.<br>• 0: Received no Palivizumab during RSV season.
Outcome	RSV related hospital admission during RSV season.
Randomisation	Not randomised, accounted for by controlling for<br>propensity of receiving treatment in the outcome model.
Start/end of follow up	Start: Beginning of first RSV season of life<br>or birth if during RSV season.<br>End: Hospital admission during RSV season, end of RSV season, 31st Dec2016<br>or death during RSV season, whichever came first.
Analysis Strategy	Average Causal Effect (ACE) of Treatment on Outcome.

Web Table 2 - Target trial protocol for case study.

2.3 Web Table 3

Disease		Eligibility criteria	Algorithm
CLD	1a	Preterm infants with moderate or severe bronchopulmonary dysplasia (BPD) with chronological age at the start of the RSV season and gestational age as follows:<br>• Aged <1.5 month & born at <34 week<br>• Aged 1.5-3 months & born at <32 week<br>• Aged 3-6 months & born <28 week<br>• Aged 6-9 months & born at <24 week	Children with ICD-10 diagnostic code P27 *"chronic respiratory disease originating in the neonatal period"* recorded anywhere in baby's record at age <9 months.
CHD	2a	Preterm infants with haemodynamically significant acyanotic CHD at the chronological ages at the start of the RSV season and gestational ages at birth:<br>• Aged <1.5 month & born at <32 week<br>• Aged 1.5-3 months & born at <30 week<br>• Aged 3-6 months & born <26 week	Aged <6 months with ICD-10 code:<br>• Q20 *"Congenital malformations of cardiac chambers & connections"*<br>• Q21 *"Congenital malformations of cardiac septa"*<br>• Q22 *"Congenital malformations of pulmonary and tricuspid valves"*<br>• Q23 *"Congenital malformations of aortic and mitral valves"*<br>• Q24 *"Other congenital' malformations of heart"*<br>• Q25 *"Congenital malformations" of great arteries"*<br>• Q26 *"Congenital malformations" of great veins"*<br><br>children aged <6 months with OPCS procedure codes:<br>• Chapter K (K01-K99) *"Heart"*<br>• Selected codes from chapter L *"Arteries and veins"* (L01-L29, L65-L83)
CLD: chronic lung disease; CHD: congenital heart disease			

Web Table 3 - The subset of eligibility criteria for Palivizumab treatment in England used in this paper and the algorithm used to identify them using electronic health records.

2.4 Web Table 4

Mechanism Strategy		α	γ	Bias	Coverage	RMSE	MCE
MAR							
	2	0.4	0	0.07	92.8	0.14	0.00
	2 (IPW)	0.4	0	0.05	92.6	0.15	0.00
	3	0.4	0	-0.01	97.6	0.22	0.01
	3 $(m = 5)$	0.4	0	-0.01	96.0	0.21	0.01
MNAR							
	2	0.4	0.4	0.17	73.0	0.22	0.00
	2 (IPW)	0.4	0.4	0.15	78.0	0.20	0.01
	3	0.4	0.4	-0.00	97.3	0.20	0.01
	3 $(m = 5)$	0.4	0.4	-0.01	95.4	0.19	0.01
	3 (MAR)	0.4	0.4	0.08	96.5	0.23	0.01
	3 (MAR)	-0.4	-0.4	-0.11	97.6	0.31	0.01

Web table 4: Simulation results for alternative strategies with $\mu = 0$. IPW is labeled strategy 2 (IPW), compared to strategy 2, and MI is labeled as 3 $(m = 5)$, which is compared to the original strategy 3 from Table 2 Section 4. **Note that $ACE^{I_E=1}$ was calculated from a single simulation with $n = 1,000,000$ and was estimated at 2.386.**
Coverage: coverage for the 95% confidence interval; RMSE:Root mean square error; MCE: Monte Carlo error.

3 Web Figures

3.1 Web Figure 1

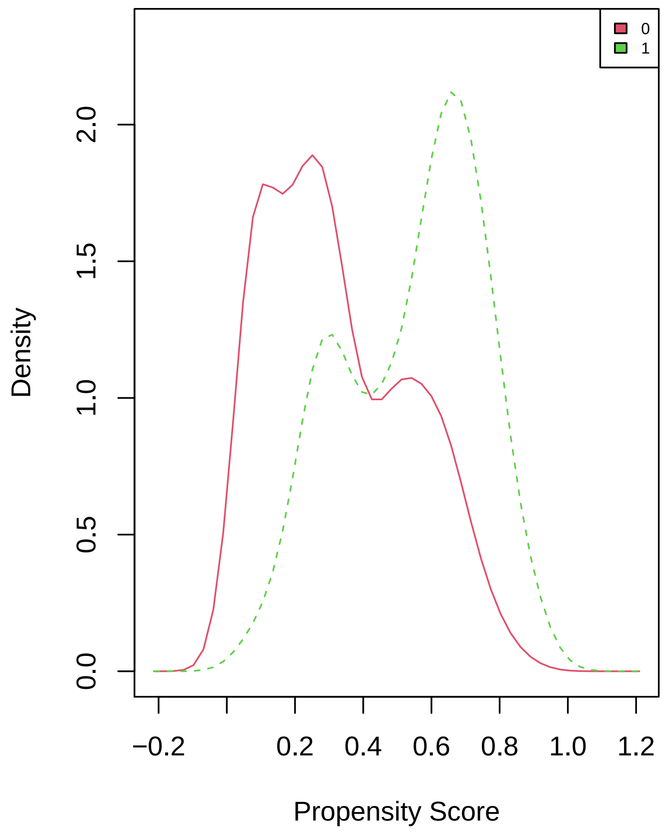

Web Figure 1: Distribution of Propensity scores for treated $(= 1)$ and untreated $(= 0)$ infants in TT_{obs} for the case study. There is reasonable enough overlap to expect propensity scores and inverse probability weights to be informative. Though there is a clear distinction in the distribution of propensity scores between treated and untreated individuals.

References

[1] Seaman Shaun, White Ian. Review of inverse probability weighting for dealing with missing data *Statistical methods in medical research.* 2013;22:278-295.
